# Midterm Results of the Open and Minimally Invasive *Transversus Abdominis* Release Technique for the Treatment of Abdominal Wall Hernias in an Academic Center

**DOI:** 10.3389/jaws.2022.10407

**Published:** 2022-08-05

**Authors:** Nicolás Quezada, Milenko Grimoldi, Ioram Jacubovsky, Nicolás Besser, Sergio Riveros, Pablo Achurra, Fernando Crovari

**Affiliations:** ^1^ Department of Digestive Surgery, Surgery Division, Faculty of Medicine, Pontificia Universidad Católica de Chile, Santiago, Chile; ^2^ General Surgery Service, Hospital Dr. Sótero Del Río, Santiago, Chile; ^3^ Surgery Division, Faculty of Medicine, Pontificia Universidad Católica de Chile, Santiago, Chile

**Keywords:** incisional hernia, giant hernia, transversus abdominis release, TAR, retro-muscular

## Abstract

**Introduction:** Large hernia defects are a challenge for general and specialized hernia surgeons. The *transversus abdominis* release (TAR) technique has revolutionized the treatment of complex hernias since it allows the closure of large midline hernias, as well as hernias in different locations. This study aims to report the experience with the TAR technique and mid-term results in the first 101 patients.

**Methods:** Non-concurrent cohort review of our prospectively collected electronic database. All patients submitted to a TAR (open or minimally invasive eTEP-TAR) from 2017 to 2020 were included. Demographic data, comorbidities, hernia characteristics, preoperative optimization, intraoperative variables, and clinical outcomes were gathered. The main outcomes of this study are hernia recurrences and surgical morbidity.

**Results:** A total of 101 patients were identified. The median follow-up was 26 months. Mean age and body mass index was 63 years and 31.4 Kg/m^2^, respectively. Diabetes was present in 22% of patients and 43% had at least one previous hernia repair. Nineteen patients had significant loss of domain. Mean hernia size and area were 13 cm and 247 cm^2^, respectively. Ninety-six percent of cases were clean or clean-contaminated. The mean operative time was 164 min and all patients received a synthetic mesh. We diagnosed two hernia recurrences and the overall (medical and surgical) complication rate was 32%. The hernia-specific complication rate was 17%, with seven surgical site infections and seven surgical site occurrences requiring procedural interventions. Notably, weight loss was associated with a lower risk of SSOPI and reoperations.

**Conclusion:** We show an encouraging 2% of recurrences in the mid-term follow-up in the setting of clinically complex hernia repair. However, we observed a high frequency of overall and hernia-specific complications pointing to the complexity of the type of surgery itself and the patients we operated on.

## Introduction

Abdominal wall hernias are a heterogeneous disease with hernia defects from a few millimeters to massive abdominal wall defects, producing a detrimental impact on core functionality and overall quality of life [1]. The treatment of AWH consists in most cases of hernia defect closure and mesh reinforcement [2]. Particularly in midline hernias, this premise cannot be easily achieved in large hernia defects, the so-called W3 hernias of the European classification [3], or in those patients who present with loss of domain (LOD).

In cases of large hernia defects, the simple closure of the hernia and mesh reinforcement produces an unacceptably high rate of hernia recurrence due to excessive tension in the midline closure, produced by the lateral abdominal musculature. Therefore, to reduce the lateral traction of the midline, and to allow medialization of the fascial edges, the component separation techniques were developed. Originally in 1990, Dr. Óscar Ramírez described the anterior component separation (ACS) which allows the medialization of the fascial edges through transection of the external oblique fascia [4].

Later, in 2012 Dr. Novitsky and others reported their initial experience in performing the posterior component separation (PCS) with *Transversus Abdominis* Release (TAR) technique, which consists in transecting the muscular and aponeurotic portion of the *transversus abdominis* muscle, allowing the medialization of the fascial edges, without compromising the skin vasculature, and placing a large mesh in the retro-muscular position [5]. Later on, the TAR technique has been described for the treatment not only of large midline hernias but also for parastomal, subxiphoid, suprapubic, lateral, post-renal, or hepatic transplantation, after open abdomen, etc. [6–9]. In 2016, Novitsky published the long-term follow-up of 428 patients repaired with the TAR technique with synthetic mesh reporting a 3.7% recurrence rate and an overall 18.7% of complications, validating the usefulness of this approach for large hernias [10]. Additionally, other reports indicate that this technique can be fully reproduced by minimally invasive surgery, either laparoscopic or robotic, although direct prospective comparisons with the open approach have not been performed yet. Anyhow, the results of the minimally invasive approach are promising in decreasing wound and systemic complications [11, 12].

Thus this paper aims to report the results of the first 101 patients operated with the TAR technique in an academic center.

## Methods

This is a non-concurrent cohort review of clinical records of our prospectively collected electronic database. The institutional review board approved this study and authorized the review of clinical charts. All patients who underwent a TAR between June 2017 and March 2020 were included. All cases were performed or assisted by the leading surgeon of this study. The TAR technique was indicated in the following scenarios: 1) when the hernia defect was wider than 10 cm according to CT scan measurements, 2) In midline hernias with associated parastomal defects, 3) in midline hernias with a lateral component, and 4) in the presence of a wide hernia with narrow rectus muscle, according to Carbonell´s rule, which predicts the need of TAR technique when the sum of both rectus muscles divided by the width of the hernia is less than 2 [13]. The surgical technique was performed as described by Novitsky and others for the open TAR [5]. Briefly, after midline incision and opening of the hernia sac and *linea alba*, a complete adhesiolysis was performed. Then, the retro rectus space was created by incising the medial border of the posterior rectus sheath (PRS), the retrorectus space is developed, carefully preserving neurovascular bundles. The posterior lamella of the internal oblique muscle was cut and the muscular and aponeurotic portion of the *transversus abdominis* muscle was incised. Then, the preperitoneal/pretransveralis plane was created by blunt dissection. Both PRS were re-approximated with running sutures of 2/0 polydioxanone. Peritoneal tears were repaired with 3/0 polyglactin sutures. Finally, a 30 × 30 cm mesh was placed in a diamond configuration in most cases and two retromuscular drainages were used. If inguinal hernias were present, dedicated meshes were placed covering each myopectineal orifices. *Linea alba* was restored with a running suture of 1/0 polydioxanone in most cases. When the tension was felt as taut, “figure of eight” interrupted stitches of 1/0 polydioxanone were placed and sequentially tied. The hernia sac was removed and the skin was closed with staples in most cases. For the video-assisted TAR, we performed an extended-view totally extraperitoneal TAR (eTEP-TAR) technique as described previously [14]. The retro rectus is created under direct camera telescopic visualization, the crossover was performed by incising the medial border of the PRS away from the hernia defect and the contralateral retro rectus space was created. The eTEP-TAR technique was performed in the same way as the open technique, the PRS was closed with running 3/0 barbed sutures, and *the linea alba* was re-approximated with 1/0 barbed sutures, and the mesh was placed in a diamond configuration. Two drainages were also used in the laparoscopic approach.

Demographic data, body mass index (BMI), ASA Score, diabetes status, smoking, and immunosuppression were retrieved. Preoperative optimization was indicated in patients with obesity (BMI higher than 30 Kg/m2) to achieve weight loss. All patients who smoked were advised to stop smoking for 4 weeks before surgery and diabetic patients were evaluated by an endocrinologist to achieve an HbA1c lower than 7.5%. Additionally, when hernia defects were wider than 18 cm, *Botulinum* toxin type A (BTA) was injected 1 month before surgery; most cases received 50 units of BTA per side by three injection spots. Loss of domain (LOD) was defined as a ratio of the volume of the hernia sac to the volume of the abdominal cavity greater than 0.25 as described by Tanaka et al. [15, 16]. When the LOD ratio was higher than 0.5, especially when associated with a hernia defect wider than 15 cm, preoperative progressive pneumoperitoneum (PPP) was added to BTA, insufflating 1000 cc per day during 5–7 days. In patients with an LOD ratio below 0.5, weight loss and BTA were used as preoperative optimization.

Hernia characteristics were described according to the EHS classification of incisional abdominal wall hernias. Additionally, the number of previous repair and operation details regarding the type, size, and weight of meshes, wound classification, concomitant surgical procedures, type of approach (open or e-TEP), and operative time were retrieved.

The main outcomes of this study are recurrences and morbidity. Recurrences were determined by abdominopelvic CT scan, clinical evaluation, or a validated telephone interview in cases where we could not reach a clinical evaluation due to the COVID-19 pandemic [17]. Of note, all patients were advised to have an abdominopelvic CT scan 12 months after surgery. Secondary outcomes are comparisons between the open and eTEP-TAR techniques, and to assess associations between clinical variables, surgical approach, and hernia-related complications.

Hernia-specific morbidity was described as surgical site occurrences (SSO), surgical site infections (SSI), and SSO requiring procedural interventions (SSOPI) as previously described [18]. Additionally, morbidity was classified according to the Clavien-Dindo classification [19].

Results are expressed as % for categorical variables and mean ± standard deviation (SD) or median with interquartile range (IQR) for numerical variables depending on data distribution. Statistical analyses were performed with the Chi-Squared test, non-paired Student t-test, or Mann-Whitney test depending on data distribution. Multivariate analysis was performed by the multi-multiple correlation analysis technique (CAT) since it allows obtaining a good graphic representation of the relationship between independent variables and outcomes. To discard confounding factors associated with main outcomes, logistic regression was performed as needed. Statistical analyses were performed with the SPSS software (Statistical Package for Social Sciences for Windows, Version 22.0, Armonk, NY, United States: IBM Corp) and statistical differences and correlations were considered significant when *p* < 0.05.

## Results

In total 101 patients underwent a hernia repair with the TAR technique between June 2017 and March 2020. Eighty-nine patients were performed by open surgery and 12 by eTEP -TAR. The whole series median follow-up is 26 months (IQR 21–32). The demographic characteristics of the series are described in Table 1. Briefly, 57 patients were female, mean BMI was 31.4 ± 6.1 Kg/m2, and a median ASA Score was 2. Regarding comorbidities, 54 (53.4%) patients had at least one, being the most frequent obesity (61.3%), active smoking (22.8%), and type-2 diabetes (21.8%). Forty-three patients had at least one previous mesh hernia repair. Nine of these patients presented infection in the previous hernia repair (8.9%).

**TABLE 1:** Demographic data.

	Whole Series	Open	eTEP-TAR
Number of patients (n)	101	89	12
Age (years, mean ± SD)	63.81 ± 12	64.2 ± 11	60.9 ± 13
Gender (male/female)	44/57	38/51	6/6
BMI (kg/m2, mean ± SD)	31.4 ± 6.1	31.4 ± 5.8	31.3 ± 8
BMI <30	39 (38.7%)	32 (36%)	7 (58.3%)
BMI 30–34.9	38 (37.6%)	36 (40.5%)	2 (16.7%)
BMI 35–39.9	16 (15.8%)	16 (17.9%)	0
BMI >40	8 (7.9%)	5 (5.6%)	3 (25%)
ASA Class (n, %)			
ASA 1	6 (6%)	6 (6.7%)	0
ASA 2	85 (84.2%)	74 (83.2%)	11 (91.6%)
ASA 3	10 (9.8%)	9 (10.1%)	1 (8.4%)
Comorbidities (n, %)			
Type-2 Diabetes mellitus	22 (21.8%)	20 (22.4%)	2 (16.6%)
Insulin resistance	16 (15.8%)	12 (13.4%)	4 (33.3%)
COPD	2 (2%)	2 (2.2%)	0
Active smoker^a^	23 (22.8%)	21 (23.5%)	2 (16.6%)
Anticoagulation	9 (8.9%)	8 (9%)	1 (8.4%)
Immunosuppression	5 (4.9%)	5 (5.6%)	0
CEDAR Score	25.4%	25.3%	25.5%
Loss of Domain			
Less than 50%	15 (14.8%)	15 (16.8%)	0
More than 50%	4 (3.9%)	4 (4.4%)	0
Preoperative Optimization			
- According %TBWL			
3–5% TBWL	6 (5.9%)	6 (5.6%)	0
5–10% TBWL	19 (18.8%)	19 (21.3%)	0
>10% TBWL	20 (19.8%)	19 (21.3%)	1
Bariatric surgery	5 (4.9%)	5 (5.5%)	0
Botulinum toxin	19 (18.8%)	19 (32.3%)	0
Progressive Pneumoperitoneum	4 (3.9%)	4 (4.4%)	0

ASA, American Society of Anesthesiologist Classification. COPD, chronic obstructive pulmonary disease.

BMI: body mass index. TBWL: total body weight loss.

a All patients were advised to stop smoking 4 weeks before surgery.

*Denotes significant differences between Open approach and eTEP-TAR groups (Chi-squared, t-Student test or Mann-Whitney depending on data type, *p* < 0.05).

Hernia characteristics are described according to the EHS classification of ventral hernias Table 2. The mean defect diameter was 13.2 ± 4 cm. Eight patients required intestinal resection (five small bowel and three colonic resections). Five small bowel and one colonic resection were performed due to bowel damage secondary to intense adhesion to previous intraperitoneal meshes, and two due to colonic enterocutaneous fistula resection (Table 3).

**TABLE 2:** Hernia characteristics and EHS classification.

	Whole Series	Open	eTEP-TAR
Recurrent hernia, n (%)	43 (42.5%)	39 (43.8%)	4 (33.3%)
Previous infection, n (%)	9 (8.9%)	6 (6.7%)	3 (25%)*
Mean hernia size			
Width, cm, (mean ± SD)	13.24 ± 4	13.6 ± 4.1	10.4 ± 1.6*
Area, cm2, (mean ± SD)	247.75 ± 149.3	263.1 ± 152	134.5 ± 36.1*
Midline hernia location (*n*, %)			
M1	5 (5%)	5 (5.6%)	0
M2	75 (74.3%)	68 (76.4%)	7 (58.3%)
M3	97 (96%)	85 (95.5%)	12 (100%)
M4	69 (68.3%)	66 (74.1%)	3 (25%)*
M5	15 (14.9%)	15 (16.8%)	0
Lateral associated defect (*n*, %)			
L1	2 (2%)	1 (1.1%)	1 (8.3%)
L2	3 (2.9%)	3 (3.3%)	1 (8.3%)
L3	10 (9.9%)	7 (7.8%)	2 (16.6%)
L4	1 (1%)	1 (1.1%)	0

*Denotes significant differences between Open approach and eTEP-TAR groups (Chi-squared, t-Student test or Mann-Whitney depending on data type, *p* < 0.05).

**TABLE 3:** Surgical results, mesh characteristics and complications.

	Whole series	Open	eTEP-TAR
	*n* = 101	*n* = 89	*n* = 12
CDC Wound Classification (*n*, %)			
Clean	70 (69.3%)	59 (66.2%)	11 (91.6%)
Clean-contaminated	27 (26.7%)	26 (29.2%)	1 (8.3%)
Contaminated	4 (4%)	4 (4.5%)	0
Dirty	0	0	0
Concomitant Surgical Procedure (*n*, %)			
Inguinal hernia	27 (26.7%)	22 (24.7%)	5 (41.6%)
Lateral hernia (EHS L1-L4)	16 (15.8%)	12 (13.4%)	4 (16%)
Cholecystectomy	14 (13.8%)	13 (14.6%)	1 (8.3%)
Mesh and tackers removal	3 (3%)	2 (2.2%)	1 (8.3%)
Intestinal resection	8 (7.9%)	8 (8.9%)	0
Full thickness intestinal injury	2 (1.9%)	2 (2.2%)	0
Operative time (min, mean ± SD)	164.7 ± 61.4	160.9 ± 61	193.3 ± 53
Mesh Characteristics			
Mesh area, cm^2^ (mean ± SD)	945.2 ± 203.7	948 ± 203	917 ± 215
Mesh weight (*n*, %)			
Lightweight	1 (0.9%)	1 (1.15%)	0
Medium weight	80 (79.1%)	68 (76.35%)	12 (100%)
Heavy weight	20 (20%)	20 (22.5%)	0
Mesh material (*n*, %)			
Polypropylene	96 (95%)	85 (95.5%)	11 (91.7%)
Polyvinylidene Fluoride (PVDF)	5 (4.9%)	4 (4.5%)	1 (8.3%)
Wound complications (*n*, %)			
SSO	17 (16.8%)	12 (13.4%)	5 (41.6%)*
SSOPI	7 (6.9%)	4 (4.5%)	3 (25%)*
SSI	7 (6.9%)	5 (5.6%)	2 (16.6%)
Mesh removal	2 (1.9%)	1 (1.1%)	1 (8.2%)
Intraoperative complications	2 (1.9%)	2 (2.2%)	0
Systemic complications	10 (9.9%)	9 (10.1%)	1 (8.3%)
Postoperative ileus	9 (8.9%)	9 (10.1%)	0
Clavien Dindo complication			
Type I	16 (15.8%)	14 (15.7%)	2 (16.6%)
Type II	7 (6.9%)	7 (7.8%)	0
Type III	7 (6.9%)	4 (3.9%)	3 (25%)
Type IV	2 (1.9%)	2 (2.2%)	0
Type V	0	0	0
Post-operative Outcomes			
Length of hospital stay, median (IQR)	5 (3–6)	5 (4–6.5)	2 (2–3)*
Reoperations	7 (6.9%)	4 (4.5%)	3 (25%)*
30-day mortality	0	0	0
Readmission	7 (6.9%)	5 (5.6%)	2 (16.6%)
Hernia recurrence	2 (1.9%)	1 (1.1%)	1 (8.3%)

SSO, surgical site occurrences; SSOPI, SSO requiring procedural interventions; SSI, surgical site infections; IQR, interquartile range.

*Denotes significant differences between Open approach and eTEP-TAR groups (Chi-squared, t-Student test or Mann-Whitney depending on data type, *p* < 0.05).

Ninety-three patients underwent a bilateral TAR, and eight patients had a unilateral TAR (One side retro-rectus and one side TAR). Only one patient did not achieve midline closure that required a small bridged area. All patients received synthetic meshes (polypropylene or PDVF) and transfascial fixation was used in 10 patients (all of the eight parastomals, one open TAR, and one eTEP-TAR) (Table 3). The mean whole series operative time was 164.7 ± 61.4 min (Table 3). The median length of stay (LOS) was 5 days (IRQ: 3–6), with a longer LOS in patients who underwent open TAR (5 days (IRQ 3–6) vs. two days (IRQ: 2–3), *p* < 0.001).

Regarding recurrences, we diagnosed two cases (1.98%) in 26 months of follow-up, one in each group, and additionally two patients in the open TAR group developed unilateral inguinal hernias.

Medical and/or surgical complications occurred in 32% of patients (Table 3). There were seven readmissions and six reoperations. Regarding specific hernia complications, there were 17 SSO (16.8%) and importantly 7 SSOPI (6.9%) which were 2 retro muscular abscesses that required mesh removal, multiple lavages, and VAC therapy. There were three SSI that required surgical lavage without mesh removal, one retro muscular hematoma, and one bedside wound drainage.

Univariate analysis demonstrated that smoking (*p* = 0.047) and eTEP-TAR (*p* = 0.014) increased the risk of having an SSO. Additionally, eTEP-TAR was associated with an increased risk of SSOPI (*p* = 0.009). Importantly, a %TBWL higher than 5% was significantly associated with a decreased risk of SSOPI (*p* = 0.03) and reoperations (*p* = 0.03). Other independent variables were not associated with SSO, SSI, or SSOPI.

Multiple Correlation Analysis Technique between independent variables (diabetes, BMI, active smoking, immunosuppression, anticoagulation, antiagregation, open vs. eTEP approach, previous repair, previous wound infection, concomitant procedure) and clinical outcomes (systemic complications, postoperative ileus, SSO, readmissions, reoperations, and length of stay) was performed. In this analysis we noticed a tendency associating active smoking and anticoagulation with SSO and reoperations. However, logistical regression did not demonstrate a statistically significant association between preoperative characteristics and clinical outcomes.

## Discussion

Patients with large hernia defects and those with loss of domain remain a challenge for general surgeons and herniologists. Several surgical approaches have been proposed to treat this complex clinical scenario with variable results in morbidity and recurrences. In this paper, we report our initial experience with mid-term results with the use of the *Transversus abdominis* release technique for the treatment of complex AWH. Here we show an encouraging 2% of recurrence with an acceptable 16.8% of hernia-specific morbidity at 26 months of follow-up.

The modern concepts of AWH treatment consider hernia defect closure and mesh reinforcement as key technical steps. Restoration of the *linea alba* provides an improvement in core stability, abdominal wall function, and quality of life [20, 21]. Extrapolating results from the IPOM repair, it has been shown that not closing the hernia defect is associated with bulging, a higher rate of hernia recurrence, worse aesthetic results, and worse functional outcomes [22]. In a series of bridged TAR repairs, the recurrence rate was as high as 46% and the quality of life and pain scores were significantly worse in those who presented hernia recurrence [23].

Mesh reinforcement has been shown to reduce recurrence rates by 50% no matter the approach selected for the hernia repair [2] or the layer of the abdominal wall selected to place the mesh. However, the best position is the retro-muscular plane since it allows faster integration and produces a scar with a higher rate of mature collagen 1 to immature collagen 3, which finally translates into a lower recurrence rate [24]. In this context, we believe that the TAR procedure offers a theoretical advantage over the ACS by placing wide meshes in the retro muscular position with a wide hernia defect overlap, without compromising the skin vasculature as needed in the ACS technique.

Additionally, as the TAR technique allows the preperitoneal dissection of the complete abdominal wall, hernias in every location can be approached by this technique. This is particularly important in complex hernias such as those near bony structures (M1 or M5 hernias) or others such as the parastomal hernias, lateral hernias, and even inguinal hernias can be repaired by the same dissection, either open or by the eTEP-TAR technique, as we did in our experience.

Although in our series the mean hernia width was 13 cm, we treated 14 patients with defects wider than 18 cm and 19 patients with significant LOD, 4 of them with LOD greater than 50%. In these complex patients, we considered the use of neoadjuvant therapy, such as PPP and botulinum toxin type A injection. Thus, 19 patients received BTA and 4 received PPP, achieving hernia defect closure in all of them, requiring prolonged postoperative intubation in one patient who had a massive 25 × 30 cm defect with 50% of LOD. It must be noted that despite our favorable results, the evidence of the use of BTA for hernia repair is still emerging and it is impossible to separate the contribution of BTA from the preoperative optimization, particularly the effect of significant weight loss.

Regarding hernia recurrences, we have documented 2 cases during the follow-up period. One occurred in a patient with an open TAR who required mesh removal due to deep infection and the other is a radiological recurrence in a patient with an eTEP-TAR who is currently asymptomatic. Fortunately, we have not observed semilunar line injuries or posterior rectus sheath dehiscence in our patients during the short and mid-term follow-up.

Medical and surgical complications occurred in a high proportion of our patients, pointing to the complexity of the operated patients as well as the complexity of the procedure itself [25]. Hernia-specific complications frequency in our series (16.8%) is comparable to others, ranging from 20 to 50% [4, 26]. Of note, we observed a significantly higher rate of SSO, SSOPI, and reoperations in the eTEP-TAR approach which a paradoxical result considering the minimally invasive nature of the procedure. When comparing the open and eTEP-TAR groups we only found that the eTEP-TAR group had a significantly lesser weight loss and a higher frequency of previously infected hernia repair, which are known risk factors for SSO and SSOPI [27–29]. However, when considering the CEDAR app score, we found no differences between groups that could explain the observed differences, making it hard to determine the reason for this increase in SSO and SSOPI in our eTEP-TAR group. However, the eTEP-TAR technique has a steep learning curve and its influence cannot be measured in our study and will be re-assessed as the number of cases increases in our institution.

An interesting result found in our experience is the effect of weight loss as a protective factor for developing SSOPI and the need for reoperations. It seems obvious that weight loss should be associated with improved clinical outcomes [28]. However, there is scarce literature indicating this association [30]. Of note, and to our best knowledge, this is the first series that statistically demonstrate an improvement in SSOPI and reoperations when patients achieved at least a 5% of TBWL. Thus, we strongly believe that weight loss should be advised to all patients, despite their initial BMI.

## Conclusion

The TAR technique is a feasible and reproducible procedure with good mid-term results in terms of recurrences and complications performed in an academic center.

## Data Availability

The original contributions presented in the study are included in the article/supplementary material, further inquiries can be directed to the corresponding author.
